# A critical assessment of the *Candida* strains isolated from cigar tobacco leaves

**DOI:** 10.3389/fbioe.2023.1201957

**Published:** 2023-08-25

**Authors:** Yun Jia, Wen Zhou, Zhen Yang, Quanwei Zhou, Yue Wang, Yi Liu, Yuhong Jia, Dongliang Li

**Affiliations:** ^1^ Cigar Fermentation Technology Key Laboratory of China Tobacco, China Tobacco Sichuan Industrial Co Ltd., Chengdu, Sichuan, China; ^2^ Industry Efficient Utilization to Domestic Cigar Tobacco Key Laboratory of Sichuan Province, China Tobacco Sichuan Industrial Co Ltd., Shifang, Sichuan, China

**Keywords:** cigar fermentation, *Candida* strains, isolation and screening, flavor components, sensory evaluation

## Abstract

**Introduction:** *Candida* genus plays a crucial role in cigar fermentation, and strains from different sources might have differences in metabolic characteristics. Therefore, this study conducted directional isolation of *Candida* strains from cigar tobacco leaves and compared their fermentabilities to screen suitable strains for cigar fermentation, thereby improving the cigar quality.

**Methods:** First, the *Candida* strains from cigars tobacco leaves in different production areas were directionally isolated by pure culture. Then, the isolated strains were screened based on chemical indexes and flavor component contents. Finally, the fermentabilities of preferred strains were verified by sensory evaluation.

**Results:** Five strains of *C. parapsilosis* and four strains of *C. metapsilosis* were obtained through directional isolation. By comparing the physicochemical indexes of nine strains of *Candida*, it was found that *C. parapsilosis* P1 and *C. metapsilosis* M4 not only reduced the alkaloids content (by 25.3% and 32.6%, respectively) but also increased the flavor components content (by 25.2% and 18.9%, respectively). Among them, P1 could raise the content of chlorophyll degradation products, carotenoid degradation products, and Maillard reaction products, and enhance the beany and nutty flavor of cigars. M4 could raise the content of chlorophyll degradation products, cembranoids degradation products, and Maillard reaction products, and improve the baking, nutty, cocoa, and honey flavor of the cigar.

**Discussion:** In this study, the *Candida* strains were directionally isolated from cigars tobacco leaves in different production areas, and two functional strains suitable for cigar fermentation were screened based on physicochemical indexes and sensory evaluation, which would contribute to the directed regulation of cigar quality and flavor diversification.

## 1 Introduction

Cigars are tobacco products rolled from tobacco leaves that have been air-dried, fermented and aged (Frankenburg, 1950). There are problems with the air-dried tobacco, such as great irritation, rough smoke, and lacking of flavor richness, and they must be fermented before they can be used to roll manufactured cigars. Fermentation could effectively degrade macromolecules such as proteins and alkaloids in tobacco leaves, reduce irritation, produce flavor components (e.g., solanone, megastigmatrienone), and ultimately forming the unique style characteristics of cigars (Li et al., 2020; Zheng et al., 2022a). In addition, the style characteristics of cigars are affected by geographic origins, climates, altitudes, species, and cultivation techniques, and various factors together lead to the different flavors and experiences of cigars (Zheng et al., 2022a).

Traditionally, tobacco fermentation is a multispecies mixed fermentation conducted in an open environment, relying on autochthonous microbes in the tobacco leaves and fermentation environment for enzymatic hydrolysis and microbial fermentation at natural conditions, that is, natural fermentation. However, long production cycles, uncertain external environment (such as seasons) and unstable quality have limited the development of the cigar industry (J. Li et al., 2020; L. Zhang et al., 2013). To improve the stability and controllability of tobacco quality, the treatment of special tobacco leaves uses artificial fermentation, including the use of steam, pressure, and high temperature to control appropriate fermentation conditions, as well as the addition of additives (such as microbial starters and spices) for fermentation. Among them, bioaugmentation fermentation, as an artificial technique to improve the quality of fermented products by adding microbial strains with specific functions, has good application effects in degrading macromolecular substances and improving product flavor (J. Liu et al., 2015; Zheng et al., 2022b)

By dissecting the complex structures and succession patterns of microbial communities during cigar fermentation, it is helpful to mine functional microbes for bioaugmentation fermentation. Since the theory of microbial fermentation of tobacco was proposed by Suchsland in 1891, numerous studies based on pure culture, denaturing gradient gel electrophoresis (DGGE) and high-throughput sequencing (HTS) technologies have been carried out to preliminarily resolve the microbial communities present in fermented tobacco products (Li et al., 2020; Zheng et al., 2022a). For example, Zheng et al. revealed microbial communities from four famous cigar-producing regions in Dominican Republic, Brazil, Indonesia, and China through HTS, and revealed that microbial communities in cigar leaves were closely related to flavor components and style characteristics (Zheng et al., 2022a). The statistical method of correlation provides an opportunity to analyze the function of complex microbial communities (Vilanova and Porcar, 2016; Wang et al., 2019), as Li et al. (M. Li et al., 2023) identified the core microbes in fermented pepper paste by analyzing the correlation between microorganisms and aroma substances. Furthermore, functional microbes could be isolated and studied for their effect on the flavor of fermented products by pure culture and bioaugmentation. For example, (Zheng et al., 2022b) significantly increased the levels of solanone, trimethyl pyrazine, and megastigmatrienone in cigar tobacco leaves by inoculation with *Acinetobacter* strains.

A previous study has shown that the *Candida* genus has the functional potential to degrade nitrogen-containing substances and synthesize flavor substances during cigar fermentation (Jia et al., 2023). However, *Candida* strains with different metabolic characteristics could cause cigars to have different sensory profiles. Therefore, this study aimed to directionally isolate *Candida* strains from cigars tobacco leaves in different production areas by pure culture method. Then, the fermentabilities of isolated strains were evaluated and compared, from which functional strains suitable for the fermentation of cigar tobacco leaves were screened. The research results would guide the directed regulation of cigar quality and diversification of style characteristics.

## 2 Material and methods

### 2.1 Materials and reagents

The fermented tobacco leaves from the Dominican Republic, Brazil, Indonesia, and China were collected and provided by China Tobacco Sichuan Industrial Co., Ltd. (Sichuan, China). Potato dextrose agar (PDA), potato dextrose broth (PDB), and yeast extract peptone dextrose agar (YPD) medium were purchased from Solarbio Technology Co., Ltd. (Beijing, China). Ampicillin sodium was purchased from Sangon Biotech Co., Ltd. (Shanghai, China). The fungal DNA kit was purchased from Tiangen Biotech Co., Ltd. (Beijing, China). High-Performance Liquid Chromatography (HPLC) grade reagents used for chromatography were obtained from Thermo Fisher Scientific (Waltham, MA, United States). Phenylethyl acetate was purchased from Sigma-Aldrich Co. Ltd. (St. Louis, MO, United States). The QuEChERS extraction kit (Kit contents: 4 g MgSO_4_, 1 g NaCl, 1 g NaCitrate, 0.5 g disodium citrate sesquihydrate, 50 mL tubes with ceramic homogenizers) was purchased from Agilent (Santa Clara, CA, United States).

### 2.2 Isolation and evaluation of *Candida* strains

To isolate *Candida* strains, the fermented tobacco leaves were mixed with a sterile saline solution and homogenized. The suspension was then gradient diluted and spread on the surface of YPD and PDA plates added with ampicillin sodium (inhibiting bacterial growth) and propionate sodium (inhibiting fungal growth), and the plates were incubated at 30°C for 3 days. The genomic DNA of isolated strains was extracted using a fungal DNA kit. The internal transcribed spacer region was amplified with primers ITS1 (5′-TCC​GTA​GGT​GAA​CCT​GCG​G-3′) and ITS4 (5′-TCC​TCC​GCT​TAT​TGA​TAT​GC-3′) (White et al., 1990). The DNA sequencing of the polymerase chain reaction (PCR) products was performed by Sangon Biotech Co., Ltd. (Shanghai, China). The isolates were identified by Basic Local Alignment Search Tool (BLAST) alignment (http://www.ncbi.nlm.nih.gov/BLAST/).

Then, the isolated *Candida* strains were separately inoculated into PDB medium and grown at 30°C with shaking for 2 days, and then the cultures were centrifuged at 12000 rpm for 30 min to collect the cells. Next, the *Candida* strains were individually inoculated into 5 kg tobacco leaves with an initial cell density of 1 × 10^6^ CFU/g. Then, the tobacco leaves were evenly stacked in a constant temperature and humidity incubator at 30°C and 70% humidity for 21 days of fermentation. The non-inoculated sample was used as a control to evaluate the fermentation ability of different *Candida* strains by detecting the chemical indexes and flavor substances of the fermented tobacco leaves.

### 2.3 Physicochemical composition analysis

As described by F. Liu et al., the contents of total sugar (TS), reducing sugar (RS), total alkaloids (NIC), and total nitrogen (TN) were detected by an active pharmaceutical ingredient (API) continuous flow instrument (Liu et al., 2021). The metabolite components were extracted by QuEChERS extraction kit, and then the contents of metabolites were examined by gas chromatography-mass spectrometry (GC-MS).

Extraction step: first, 2 g of sample and 10 mL water were added to a 50 mL tube with ceramic homogenizers, and shaken until the sample was sufficiently infiltrated by water. Then, the metabolites were extracted by adding 10 mL acetonitrile and 50 μL phenylethyl acetate internal standard (9.06 mg/mL) and shaking at 2000 rpm for 2 h. After the mixture was frozen at −20°C for 10 min, a salt packet (4 g MgSO_4_, 1 g NaCl, 1 g NaCitrate, 0.5 g disodium citrate sesquihydrate) was added and immediately shaken to remove water. Finally, 0.15 g MgSO_4_ was added to 1 mL of the supernatant and shaken at 2000 rpm for 2 min. After centrifuging at 6,000 rpm for 2 min, the supernatant was collected for further analysis.

The GC-MS conditions were described by (Jia et al., 2023). Separations of the metabolites compounds were performed on a DB-5MS column (60 m × 1.0 μm × 0.25 mm). The initial temperature of the GC oven was 60°C, and ramped to 250°C at 2 C/min, then ramped to 290°C at 5°C/min, finally held for 20 min at final temperature. Helium was used as the carrier gas at the flow rate of 1.2 mL/min. MS was operated in the electron impact mode with an ion source temperature of 230°C and an ionization voltage of 70 eV. Mass scan range was 26–400 atomic mass units. Qualitative analysis of compounds was performed by matching the mass spectra with the National Institute of Standards and Technology and Wiley Library. Compounds quantification was calculated according to the ratio between the peak area of a particular compound and the internal standard.

### 2.4 Sensory quality evaluation

After fermentation, the tobacco leaves were rolled into cigarettes with a length of 110 mm and a diameter of 14 mm. Then, the cigarette was conserved for 1 month in a constant temperature and humidity incubator with a temperature of 20°C and relative humidity of 60% for smoking evaluation. The sensory quality evaluation was performed following the Standard Evaluation Form provided by Great Wall Cigar Factory (Sichuan, China) (Hu et al., 2022). The quality characteristics consisted of mellowness, richness, matureness, irritation, smoothness, fluentness, sweetness, cleanliness, aftertaste, combustibility, ash color, and ash coagulation. The flavor characteristics mainly included caramel, beany, baking, nutty, cocoa, honey, resinous, hay, leather, and pepper flavor. A well-trained sensory panel consisting of 10 assessors majoring in cigar production was invited for the sensory quality assessment.

### 2.5 Statistical analysis

The Spearman’s pairwise correlations and the significance of the correlations were calculated using corr. test function and the psych package in R (Version R-3.5.1). Significance difference analysis and Z-score normalization were performed using SPSS (Version 22.0, SPSS Inc., Chicago, IL, United States). Further statistical analysis and graphics were performed in EXCEL 2017 software (Microsoft Office, United States) and GraphPad Prism Software (Version 8.0, GraphPad Software, San Diego, California, United States).

## 3 Results

### 3.1 Isolation of *Candida* strains

In a previous study, correlations between physicochemical metabolites and microbial communities during cigar fermentation have been analyzed (Jia et al., 2023). Correlation results indicated that the *Candida* genus was significantly negatively correlated with total nitrogen and alkaloids contents, while positively correlated with the total contents of flavor components, which implied that the *Candida* genus had the functional potential to degrade nitrogen-containing substances and synthesize flavor substances during the cigar fermentation (Supplementary Table S1). Therefore, this study conducted directional isolation of *Candida* strains from cigar tobacco leaves collected from the Dominican Republic, Brazil, Indonesia, and China. As shown in Table 1, a total of 4 strains of *C. metapsilosis* and 5 strains of *C. parapsilosis* were isolated. Next, 9 strains of *Candida* were separately inoculated into cigar tobacco leaves with an initial cell density of 1 × 10^6^ CFU/g, and the physicochemical indexes of the fermented tobacco leaves were detected and analyzed.

**TABLE 1:** Identification results of *Candida* isolates.

Isolates	Identification	Source of isolation
M1	*C. metapsilosis*	Indonesia
M2	*C. metapsilosis*	Dominican Republic
M3	*C. metapsilosis*	China
M4	*C. metapsilosis*	China
P1	*C. parapsilosis*	China
P2	*C. parapsilosis*	Brazil
P3	*C. parapsilosis*	China
P4	*C. parapsilosis*	Indonesia
P5	*C. parapsilosis*	China

### 3.2 Comparison of chemical indexes

**FIGURE 1:**
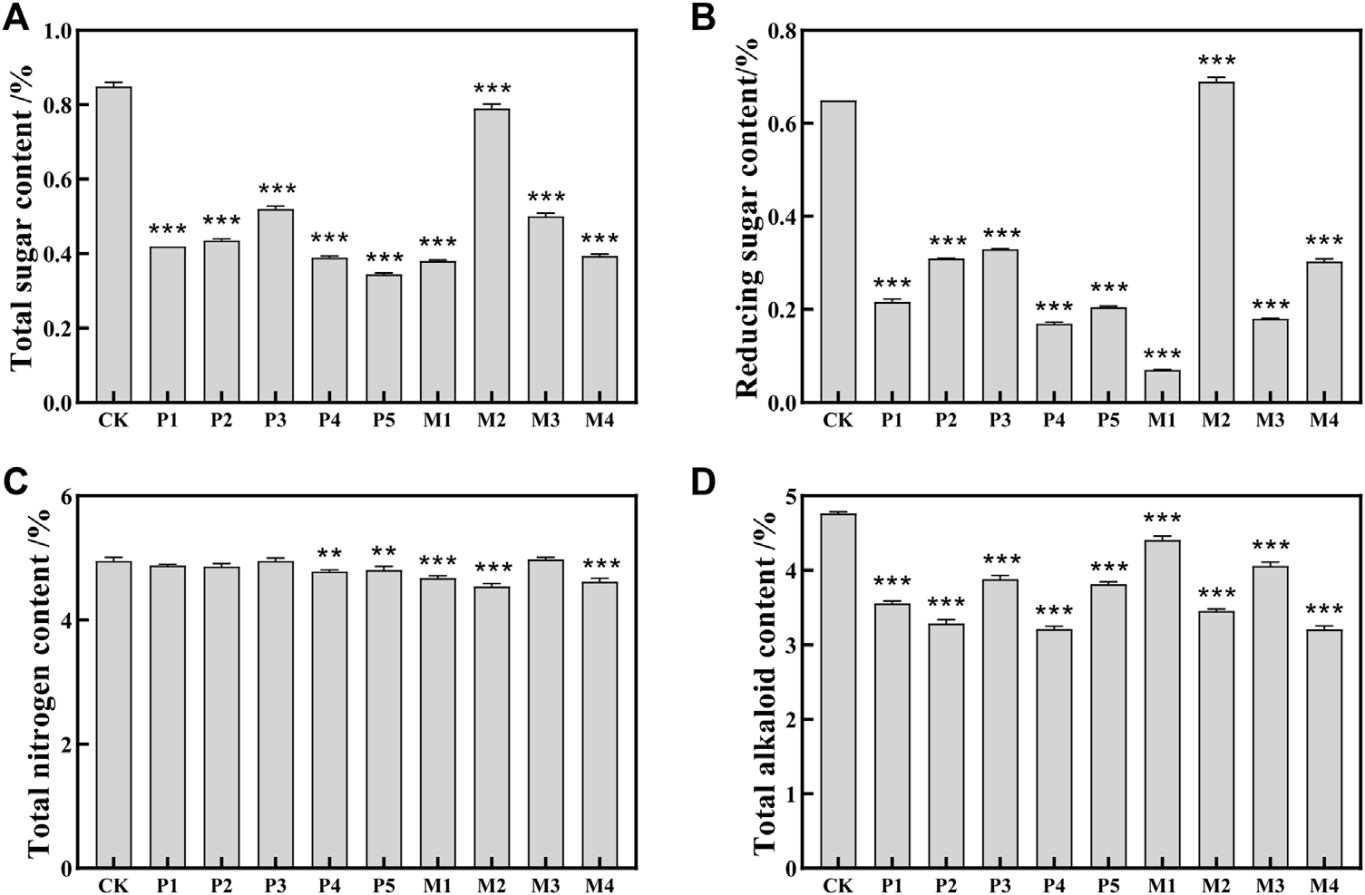
The contents of chemical indexes in tobacco leaves fermented with different *Candida* strains. **(A)** total sugar content; **(B)** reducing sugar content; **(C)** total nitrogen content; **(D)** alkaloids content. CK represented the control group, P1∼P5 represented different *C. parapsilosis* fermentation groups, M1∼M4 represented different *C. metapsilosis* fermentation groups. Asterisks indicate significant differences between the control group and bioaugmentation fermentation groups (Dunnett’s test). *: *p* < 0.05; **: *p* < 0.01; ***: *p* < 0.001.

As shown in Figures 1A, B, the contents of total sugar and reducing sugar in the bioaugmentation fermentation groups (P1∼P5, M1, M3, M4) were significantly reduced by 38.8%–89.2% compared with the control group. However, compared with the control group, the total sugar content in the M2 group was decreased by 7.1%, and the content of reducing sugar was increased by 6.2%, which reflected the differences in the utilization ability of sugar compounds among different *Candida* strains (Rivera and Tyx, 2021).

The protein in cigar tobacco leaves would produce a burnt feather flavor when smoked, and its content is one of the important indicators affecting the irritancy and cleanliness of cigars. Compared with the control group, the total nitrogen contents in the M2, M4, M1, P4, and P5 groups were significantly decreased by 8.3%, 6.7%, 5.5%, 3.4%, and 2.8%, respectively (Figure 1C). In contrast to the high sugar content in the M2 group, its total nitrogen content was the lowest among all the bioaugmentation groups, indicating that the M2 strain might have a higher utilization capacity for nitrogen-containing substances such as proteins.

In addition, higher alkaloids content could not only cause bitterness and irritancy of tobacco leaves but also have potential addictive and carcinogenic effects on humans (Kaminski et al., 2020; Rehder Silinski et al., 2020). As shown in Figure 1D, the alkaloids contents in all bioaugmentation groups showed a significant decrease compared to the control group. The order of alkaloids content in these 10 groups from low to high was: M4 < P4 < P2 < M2 < P1 < P5 < P3 < M3 < M1 < CK. Compared with control group, the alkaloids contents of M4, P4, P2, M2, and P1 groups decreased by 32.6%, 32.5%, 30.9%, 27.5%, and 25.3%, respectively.

**FIGURE 2:**
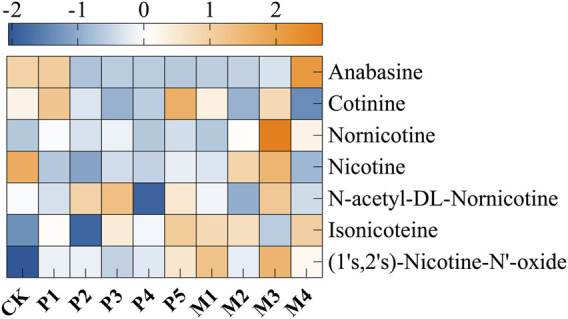
The changes in the content of nicotine and degradation products in different fermentation groups. CK represented the control group, P1∼P5 represented different *C. parapsilosis* fermentation groups, M1∼M4 represented different *C. metapsilosis* fermentation group.

Furthermore, qualitative and quantitative analysis of different alkaloids in tobacco leaves were conducted by GC-MS, and a total of seven alkaloids were detected, including cotinine, nornicotine, nicotine, N-acetyl-DL-nornicotine, isonicoteine, (1′s, 2′s)-nicotine-N′-oxide (Figure 2). Among them, nicotine accounted for 94.2–98.8% of the alkaloids content and was the most dominant alkaloids. The nicotine contents of these 10 groups were ranked from low to high as: P2 < M4 < P1 < P4 < P3 < M1 < P5 < M2 < M3. Compared with the control group, the nicotine content of the P2, M4, and P1 groups decreased by 56.3%, 51.8%, and 47.6%, respectively. In general, the alkaloids contents were significantly decreased after bioaugmentation with the P1, P2, P4, and M4 strains, and the contents of seven alkaloids in the M4 group were lower than those in other bioaugmentation groups. This might be due to the conversion of alkaloids such as nicotine to amino acids, organic acids, and volatile ammonia by *Candida* strains during fermentation.

### 3.3 Comparison of flavor components

**FIGURE 3:**
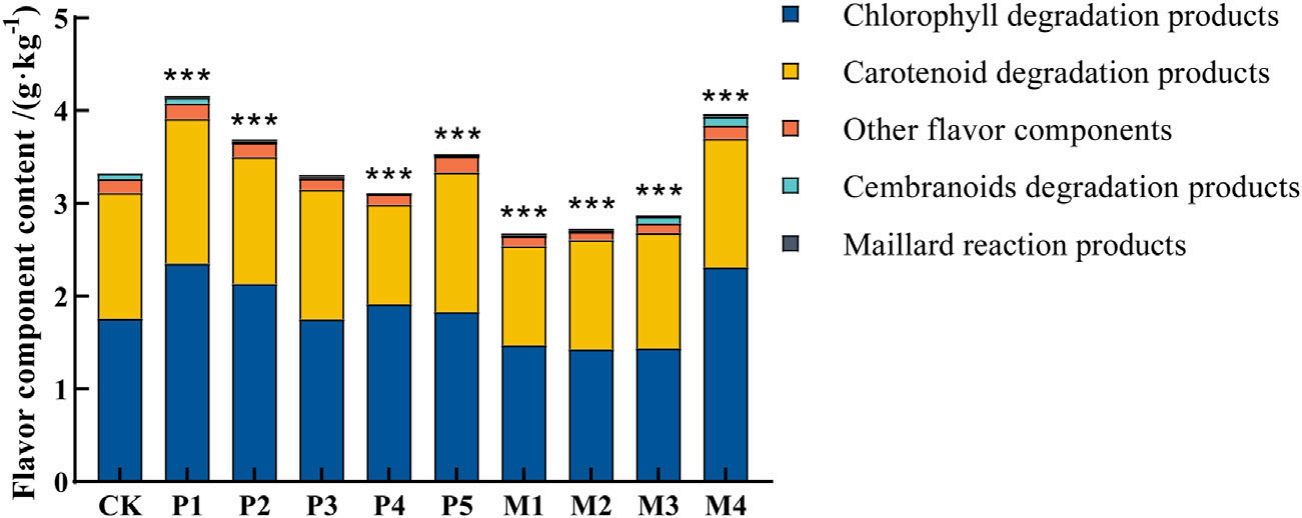
Flavor component content in tobacco leaves fermented with different *Candida* strains. CK represented the control group, P1∼P5 represented different *C. parapsilosis* fermentation groups, M1∼M4 represented different *C. metapsilosis* fermentation groups. Asterisks indicate significant differences between the control group and bioaugmentation fermentation groups (Dunnett’s test). *: *p* < 0.05; **: *p* < 0.01; ***: *p* < 0.001.

Previous studies have shown that the total content of flavor components is an important indicator of the quality of cigar tobacco leaves (Banozic et al., 2021; Hu et al., 2022; Li et al., 2020). According to the classification of precursors, flavor components were mainly classified into chlorophyll degradation products, carotenoid degradation products, Maillard reaction products, cembranoids degradation products, and other flavor components (Figure 3). The total contents of flavor components of cigar leaves after bioaugmentation fermentation by different *Candida* strains were ranked from high to low as follows: P1 > M4 > P2 > P5 > P3 > CK > P4 > P5 > M3 > M2 > M1. Compared with the control group, the total content of flavor components in the P1, M4, P2, and P5 groups significantly increased by 25.2%, 18.9%, 11.1%, and 6.0%, respectively, while those in M1, M2, M3, and P4 groups significantly decreased by 19.5%, 17.7%, 13.5%, and 6.6%, respectively. The content of flavor components in tobacco leaves after bioaugmentation with different strains suggested that P1 and M4 strains might have higher abilities to synthesize flavor substances.

**FIGURE 4:**
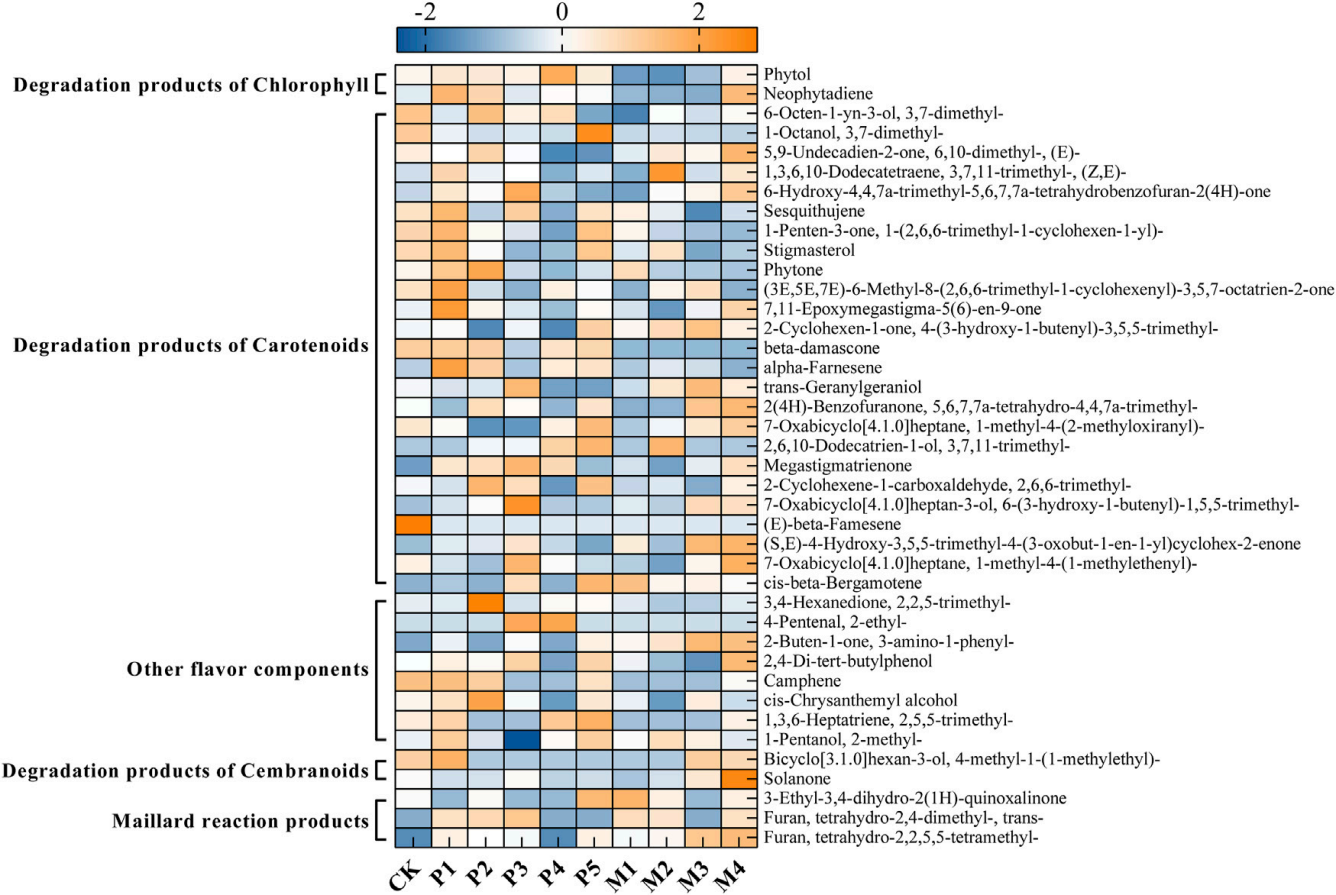
The changes in the content of flavor substances in different fermentation groups. CK represented the control group, P1∼P5 represented different *C. parapsilosis* fermentation groups, M1∼M4 represented different *C. metapsilosis* fermentation group.

Figure 4 showed the changes in the content of 34 flavor substances in different fermentation groups, with the majority of flavor components in the P1 and M4 groups being higher than in the other bioaugmentation groups. Compared with the control group, 25 and 21 flavor components were found to be higher in the P1 and M4 groups, respectively. In group P1, chlorophyll degradation products (e.g., phytol, neopphytodiene) and carotenoid degradation products (e.g., 1- (2,6,6-trimethyl-1-cyclohexen-1-yl)-1-Penten-3-one, phytone, α-farnesene, megastigmatrienone) were more abundant than those in the control group (Table 2). In the group M4, the contents of chlorophyll degradation products (e.g., neophytodiene), carotenoid degradation products (e.g., 6,10-dimethyl-5,9-undecadien-2-one, trans-geranylgeraniol, 5,6,7,7a-tetrahydro-4,4,7a-trimethyl-2 (4 h)-benzofuranone, megastigmatrienone, 1-methyl-4- (1-methylethenyl)-7-oxabicyclo [4.1.0] heptane, cis-beta-bergamotene), cembranoids degradation products (e.g., solanone), and Maillard reaction products (e.g., 3-ethyl-3,4-dihydro-2 (1H)-quinoxalinone, tetrahydro-2,4-dimethyl-furan, tetrahydro-2,2,5,5-tetramethyl-furan) were higher than those in the control group. Overall, the total contents of chlorophyll degradation products, carotenoid degradation products, and Maillard reaction products were significantly higher in the P1 group than those in the control group, while the total contents of chlorophyll degradation products, cembranoids degradation products, and Maillard reaction products were significantly higher in M4 group (Table 2).

**TABLE 2:** The contents of flavor compounds after fermentation with *Candida* strains P1 and M4 (mg/kg).

CAS	Compounds name	CK	P1	M4	Flavor^a^
150-86-7	Phytol	216.0	228.4	218.5	Floral
504-96-1	Neophytadiene	1542.5	2124.1	2096.4	Freshness
**Chlorophyll degradation products**		**1758.5**	2352.5***	2135.2**	
29171-20-8	6-octen-1-yn-3-ol, 3,7-dimethyl-	70.5	32.7	43.2	Fruity
106-21-8	1-octanol, 3,7-dimethyl-	62.8	41.6	34.4	Floral
3796-70-1	5,9-undecadien-2-one, 6,10-dimethyl-, (e)-	38.8	35.4	49.7	Fruity, floral
13741-21-4	1,3,6,10-dodecatetraene, 3,7,11-trimethyl-, (z,e)-	13.2	39.2	32.0	Fruity, herb
73410-02-3	6-hydroxy-4,4,7a-trimethyl-5,6,7,7a-tetrahydrobenzofuran-2 (4 h)-one	32.9	47.5	56.2	Fruity, musk, wood
58319-06-5	Sesquithujene	14.1	19.9	7.5	Spice, herb
127-43-5	1-penten-3-one, 1- (2,6,6-trimethyl-1-cyclohexen-1-yl)-	73.3	84.8	44.9	Floral
83-48-7	Stigmasterol	254.8	282.0	194.5	Beany
502-69-2	Phytone	69.7	82.7	56.5	Floral
17974-57-1	(3e,5e,7e)-6-methyl-8- (2,6,6-trimethyl-1-cyclohexenyl)-3,5,7-octatrien-2-one	21.6	38.9	0.0	Fruity
64243-62-5	7,11-epoxymegastigma-5 (6)-en-9-one	38.1	65.1	50.5	Fruity
34318-21-3	2-cyclohexen-1-one, 4- (3-hydroxy-1-butenyl)-3,5,5-trimethyl-	13.6	15.1	17.3	Floral, woody, nutty
35044-68-9	Beta-damascone	108.7	109.4	0.0	Fruity, rose
502-61-4	Alpha-farnesene	31.7	162.3	10.7	Fruity, herb
24034-73-9	Trans-geranylgeraniol	42.5	33.3	59.8	Spice, herb
15356-74-8	2 (4 h)-benzofuranone, 5,6,7,7a-tetrahydro-4,4,7a-trimethyl-	38.7	33.0	48.5	Fruity, musk, wood
96-08-2	7-oxabicyclo[4.1.0]heptane, 1-methyl-4- (2-methyloxiranyl)-	135.1	117.0	159.0	Fruity
38818-55-2	Megastigmatrienone	18.1	54.0	57.3	Nutty, floral, woody
432-24-6	2-cyclohexene-1-carboxaldehyde, 2,6,6-trimethyl-	148.6	137.2	164.8	Fruity
72777-88-9	7-oxabicyclo[4.1.0]heptan-3-ol, 6- (3-hydroxy-1-butenyl)-1,5,5-trimethyl-	18.2	28.0	50.0	Floral, woody
18794-84-8	(e)-beta-famesene	15.8	0.0	0.0	Spice, herb, fresh green, sweet
39763-33-2	(s,e)-4-hydroxy-3,5,5-trimethyl-4- (3-oxobut-1-en-1-yl)cyclohex-2-enone	39.8	52.4	88.9	Floral
1195-92-2	7-oxabicyclo[4.1.0]heptane, 1-methyl-4- (1-methylethenyl)-	55.1	31.3	104.8	Fruity
55123-21-2	cis-beta-bergamotene	0.0	14.2	50.9	ruity
**Carotenoid degradation products**		**1355.6**	1557.0**	**1366.7**	
96-76-4	2,4-di-tert-butylphenol	96.1	98.6	108.2	fFruity
79-92-5	Camphene	17.5	17.6	7.4	Camphor, mothball, oil, warm
18383-59-0	cis-chrysanthemyl alcohol	20.0	25.2	11.6	Floral
29548-02-5	1,3,6-heptatriene, 2,5,5-trimethyl-	16.8	24.1	15.5	Fruity
**Other flavor compounds**		**150.4**	**165.5**	**154.7**	
513-23-5	bicyclo [3.1.0] hexan-3-ol, 4-methyl-1- (1-methylethyl)-	36.2	50.0	31.8	Spice, herb
54868-48-3	Solanone	23.8	16.1	66.1	Fruity, tobacco
**Cembranoids degradation products**		**60.0**	**66.0**	16.6***	
39168-02-0	Furan, tetrahydro-2,4-dimethyl-, trans-	0.1	8.7	8.6	Nutty, sweet
15045-43-9	Furan, tetrahydro-2,2,5,5-tetramethyl-	0.0	11.7	18.9	Nutty, sweet
**Maillard reaction products**		**0.1**	20.4***	19.5***	

^a^ From flavor databases (https://www.femaflavor.org/flavor-library; http://www.thegoodscentscompany.com; https://foodb.ca/compounds).

Asterisks indicate significant differences between the control group and bioaugmentation fermentation groups (Dunnett’s test). *: *p* < 0.05; **: *p* < 0.01; ***: *p* < 0.001.

The bold words “Chlorophyll degradation products, Carotenoid degradation products, Cembranoids degradation products, Maillard reaction products, Other flavor compounds” represent the classification of different substances.

### 3.4 Sensory evaluation

**FIGURE 5:**
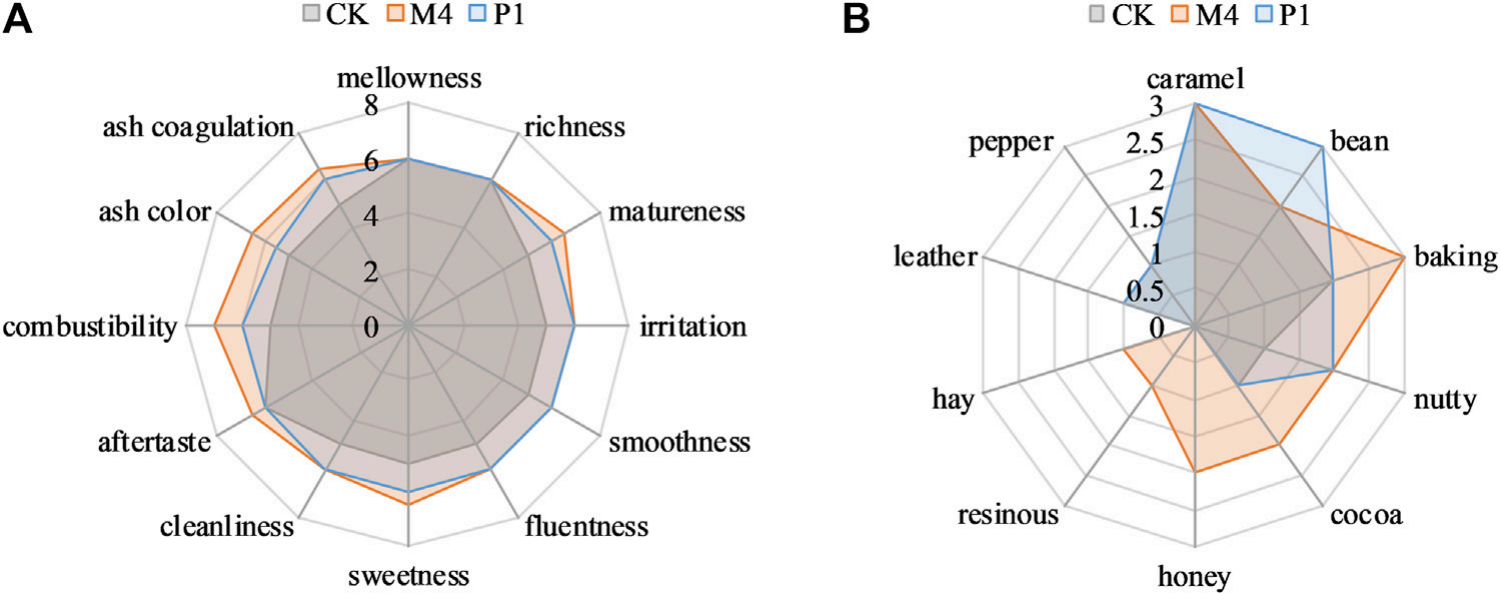
Radar plots of sensory scores of tobacco leaves fermented with *Candida* strains P1 and M4. **(A)** flavor characteristics; **(B)** quality characteristics. CK represented the control group, P1 represented P1 fermentation groups, M4 represented M4 fermentation groups.

By comparing the metabolic characteristics of nine *Candida* strains, it was found that *C. parapsilosis* P1 and *C. metapsilosis* M4 strains could not only increase the total content of flavor components but also reduce the alkaloids content in cigar tobacco leaves, which have great potential as starter. Therefore, the bioaugmentation effects of the P1 and M4 strains were verified by sensory evaluation. It was found that both P1 and M4 strains could improve the irritation and cleanliness, enhance the matureness, smoothness, fluentness, sweetness, and combustibility, and improve the coagulation and color of ash (Figure 5A). Among them, P1 could improve the beany and nutty flavor of cigar tobacco leaves, while M4 could enhance the baking, nutty, cocoa, honey, resinous, and hay flavor (Figure 5B).

## 4 Discussion

The main purpose of cigar fermentation is to enhance flavor richness by increasing the flavor component content and improve irritation by minimizing the alkaloids content of tobacco leaves (Frankenburg, 1950; Li et al., 2020). Previous correlation analyses implied that the *Candida* genus was negatively correlated with the nitrogen-containing substance contents, positively correlated with flavor components contents, and has been used in the production of traditional food fermentation (Jiang et al., 2021; Lu et al., 2021; Jia et al., 2023). For example, Bressani (Bressani et al., 2021) studied the effect of inoculation of *C. parapsilosis* on coffee and found that the body, overall flavor, aftertaste, and acidity were positively correlated with *C. parapsilosis* population. However, *Candida* strains from different sources have different properties that could lead to different sensory characteristics of cigars. Therefore, this study selectively isolated *Candida* strains from cigar tobacco leaves from the Dominican Republic, Brazil, Indonesia, and China using a pure culture method. Then, the fermentation performances of nine isolated *Candida* strains were compared based on chemical indexes and flavor components. It was found that the alkaloids content decreased and flavor components content increased to various degrees in the bioaugmentation group. This might be due to the degradation of proteins and alkaloids into small molecules amino acids and flavor substances by *Candida* strains during fermentation (Di Giacomo et al., 2007; Frankenburg, 1950; Liu et al., 2015).

Among them, *C. parapsilosis* P1 and *C. metapsilosis* M4 could significantly reduce the alkaloids content and increase the flavor components content in cigar tobacco leaves compared to other strains, which have great potential as fermentation starters. Compared with the control group, the alkaloids contents of P1 and M4 decreased by 25.3% and 32.6%, the nicotine contents decreased by 47.6% and 51.8%. Alkaloids could cause bitterness and irritancy in tobacco leaves, and reducing their content could help improve the maturity and safety of cigars (Kaminski et al., 2020; Rehder Silinski et al., 2020). In addition, the total contents of flavor components of P1 and M4 groups increased by 25.2% and 18.9%, respectively, compared with the control group. According to the classification of precursors, flavor components were mainly classified into degradation products of chlorophyll, degradation products of carotenoids, Maillard reaction products, and degradation products of cembranoids (Rodriguez-Bustamante et al., 2005). The types and content of these flavor substances had a crucial influence on the flavor profile and quality of cigar tobacco leaves. Specifically, the total contents of chlorophyll degradation products, carotenoid degradation products, and Maillard reaction products were higher in the P1 group than in the control group, while the total contents of chlorophyll degradation products, cembranoids degradation products, and Maillard reaction products were significantly higher in the M4 group. The sensory evaluation further showed that both P1 and M4 could improve irritancy and cleanliness. This might be due to the degradation of irritant proteins and alkaloids by *Candida* strains while generating chlorophyll degradation products (e.g., neophytodienes) that could increase flavor and decrease irritancy (Li et al., 2020). Among them, P1 could improve the beany and nutty flavors of cigar tobacco leaves, which might be attributed to its ability to elevate the contents of carotenoid degradation products (e.g., megastigmatrienone) and Maillard reaction products (e.g., tetrahydro-2,2,5,5-tetramethyl--furan) in cigar leaves (Zhang et al., 2021; Zhu et al., 2021). M4 could enhance the flavor of baking, nutty, cocoa, and honey, which might be related to the fact that M4 could raise the contents of carotenoid degradation products (e.g., cis-beta-bergamotene), cembranoids degradation products (*e.g.,* solanone), and Maillard reaction products (e.g., tetrahydro-2,4-dimethyl-furan) in cigar tobacco leaves (Koné et al., 2016; Zhang et al., 2013).

## 5 Conclusion

In conclusion, this study conducted targeted isolation of *Candida* strains from different sources of cigar tobacco leaves, and explored the functions of the nine isolated strains of *Candida*. Based on chemical indexes and flavor components, *C. parapsilosis* P1 and *C metapsilosis* M4 were selected as fermentation starters suitable for cigar fermentation. The bioaugmentation fermentation further verified that P1 and M4 could not only enhance the flavor richness by increasing the flavor components but also improve irritancy and safety by reducing the total nitrogen and alkaloids content. Bioaugmentation fermentation by mining and screening strains with different flavor profiles would help to highlight the typicality of cigar style characteristics and promote flavor diversification. In the future, we would further explore the effects of different addition amounts, addition timing, temperature, and relative humidity on the quality of cigar tobacco leaves, and monitor the changes of microbial community and functional genes during the bioaugmentation process by multi-omics methods such as amplicon sequencing and metagenomics, so as to better understand the fermentation mechanism of *Candida* and its impact on cigar tobacco quality.

## Data Availability

The datasets presented in this study can be found in online repositories. The names of the repository/repositories and accession number(s) can be found in the article/Supplementary Material.
