# Supplementary material for: A critical assessment of the Candida strains isolated from cigar tobacco leaves
Source: Front Bioeng Biotechnol. 2023 Aug 25;11:1201957. doi: 10.3389/fbioe.2023.1201957 (PMC10485251; doi:10.3389/fbioe.2023.1201957)
Supplement: Supplementary file 1 [file DataSheet1.PDF]

frontiers

Supplementary Material

A Critical Assessment of the *Candida* Strains Isolated from Cigar Tobacco Leaves

Yun Jia[1,2], Wen Zhou[2], Zhen Yang[1], Quanwei Zhou[1], Yue Wang[1], Yi Liu[2], Yuhong Jia[2], Dongliang Li[1,*]

*** Correspondence**

Dongliang Li: 360188228@qq.com

1 Supplementary Tables

Supplementary Table 1 Spearman Correlations between microbial genera and fermentation indexes during the fermentation

Fermentation indexes	*Aspergillus*	*Pantoea*	*Candida*	*Colletotrichum*
Total sugar	0.70**	-	-	-
Reducing sugar	0.61**	-0.61**	-	-
Total nitrogen	-	-	-0.65**	0.73***
Total alkaloids	-	-	-0.65**	0.73***
Flavor components	-	-	0.64**	-0.71**

[a]Only statistically significant correlations (statistically significant $P < 0.05$ and spearman's correlation coefficient $|\rho| > 0.6$) were shown in the table. ***, $P < 0.001$; **, $P < 0.01$; *, $P < 0.05$.
